# Heterosis Breeding in Eggplant (*Solanum melongena* L.): Gains and Provocations

**DOI:** 10.3390/plants9030403

**Published:** 2020-03-24

**Authors:** Ashish Kumar, Vinay Sharma, Bharat Taindu Jain, Prashant Kaushik

**Affiliations:** 1 National Agri-Food Biotechnology Institute (NABI), Punjab 140308, India; ku.ashish@nabi.res.in; 2 International Crops Research Institute for the Semi-Arid Tropics (ICRISAT), Hyderabad 502324, India; s.vinay@cgiar.org; 3 Department of Genetics & Plant Breeding, CCS Haryana Agriculture University, Hisar, Haryana 125001, India; jainbharat91@hau.ac.in; 4 Instituto de Conservación y Mejora de la Agrodiversidad Valenciana, Universitat Politècnica de València, 46022 Valencia, Spain; 5 Nagano University, 1088 Komaki, Ueda, Nagano 386-0031, Japan

**Keywords:** eggplant, general combining ability, heterosis prediction, epigenetic regulation, genomics

## Abstract

Heterosis (or hybrid vigor) results in a hybrid’s phenotypic superiority over its founder parents for quantitative and qualitative traits. Hybrid vigor is defined by mechanisms such as dominant complementation, over-dominance, and epistasis. Eggplant (*Solanum melongena* L.) is an essential vegetable crop and a good source of dietary minerals, vitamins, and anthocyanins, with a high oxygen radical absorbance capacity and low caloric value. Given the economic and nutritional significance of eggplants, breeding efforts focus on developing high-yielding varieties—mostly F_1_ hybrids—with important traits. Studies indicate the successful exploitation of heterosis in the eggplant for a considerable improvement with respect to quantitative traits. In this direction, estimating heterosis for yield-related traits could well be useful for examining the most beneficial hybrid mix with the exploitation of top-quality hybrid. This review examines the current perception of the breeding and molecular aspects of heterosis in eggplants and cites several studies describing the mechanisms. Rendering and combining recent genomics, epigenetic, proteomic, and metabolomics studies present new prospects towards the understanding of the regulatory events of heterosis involved in the evolution and the domestication of the eggplant ideotype.

## 1. Introduction

Eggplant, popularly known as brinjal or aubergine (*Solanum melongena* L.) is among the extensively cultivated vegetables. Consequently, it is also referred to as the common man’s vegetable in the Indian subcontinent owing to its year-around availability [1]. The cultivated area under eggplant cultivation is around 1.79 million ha with a production of 51.28 million tons [2]. Moreover, there is a continuous rise in the production of the cultivated eggplant that it has increased to 50%owing to the availability of high yielding varieties and hybrids [3]. Likewise, from the last few years, farmer’s interest and preference towards hybrids of eggplant have increased dramatically. Moreover, to overcome yield targets and to fulfil the demand, the researchers are focusing on delivering high yielding eggplant hybrids [4,5]. Heterosis is a phenomenon in which a progeny of distinct individuals exhibitshigher/lower values for the traits than the average of any of the original parent used for the development of the hybrid [6]. The value of heterosis in vegetable crops is evident from the drastic yield increases measured over the last 50 years, following the introduction of hybrids into crop production.

Hybrids and improved agronomic techniques have resulted in a steady linear increase in the performance of vegetable crops [7,8]. In the case of eggplant, first-time Kakizaki [9] found the potential use of hybrids as commercially, citing the higher yields of hybrids compared to a standard for the years 1923-1926 [10]. Further, the authors also determined that the level of heterosis is directly proportional to the diversity among the parents, which brought together several favorable diverse alleles of several genes. Thereafter several studies comforting the possibility of heterosis for every possible character have been published [11].

Heterosis has been extensively utilized in cultivated plants. In this direction, the genetic basis of heterosis has been studied for nearly a century. Two concepts are farmed as the common explanations for heterosis phenomenon dominance and over-dominance hypothesis. In the dominance hypothesis, heterosis is regarded as the result of the complementation of the deleterious alleles that were present in the inbred parental lines. Whereas, over-dominance hypothesis interpretation points out that the allelic interactions specific to the hybrid are such that the heterozygous alleles in the hybrid combination perform better than either of the homozygous ones [6,12,13]. Moreover, efforts are continuing to decode the molecular basis of heterosis correctly, but breeders continue to improve inbreds. Whereas, new technologies such as gene expression profiling are underway; efforts are being made to exploit heterosis phenomena [14].

Due to a large part of eggplant cultivation relies on cultivated varieties rather than hybrids and being an autogamous vegetable crop, pure lines are quickly developed because that the genetic base of cultivated eggplant gradually narrowed down in the course of time [15]. Similarly, hand emasculation is easy to perform owing to the large size of eggplant flowers and a successful cross can produce somewhere between 20-200 based on its genotype [3,16,17]. Moreover, male-sterility has been discovered; it is also facilitating the hybrid development in eggplant. Identification of good combiner parents is vital for hybrid development in eggplant. The combining abilities, namely general combing ability (GCA) and specific combining ability (SCA) values are critical in predicting the hybrid performance and suitability [18]. This review provides useful information concerning the heterosis for important traits in eggplant and will be a valuable resource for eggplant breeders to circumscribe the extent of heterosis for a particular trait, also presenting the genetic and molecular basis of heterosis in eggplant.

## 2. Origin, Evolution and Domestication

Eggplant (*Solanum melongena* L) is a native to the Indian subcontinent. Most of the *Solanum* species, including eggplant, are characterized by flattened seeds and curved embryos [19]. Eggplant is a berry-producing vegetable belonging to the large family of Solanaceae, which has around 3000 different species distributed in across 90 genera [20]. Out of these *Solanum* is the largest, with approximately 1500 species [21]. In a broader sense, the name ‘eggplant’ commonly meant for three species of *Solanum. Solanum melongena* L., a globally cultivated species of Asian origin. Likewise, scarlet (*S. aethiopicum* L.) and gboma (*S. macrocarpon* L.) as African eggplants. By and large, *Solanum melongena* L. is widely accepted with a primary concern because of its acreage at large scales on almost every continent. Noticeably, the wild relatives of eggplant usually have a smaller fruit size. Several forms, shape and colors of eggplant are found across Southeast Asia, indicating that this area might be the secondary center of variation. Vavilov [22] considered its center of origin in the Indo-Burma region. In another study, Vavilov [23] highlighted the “Indo-Chinese center” as the center of origin of *S. melongena*. Although, according to recent studies on the domestication of eggplants, still there are several unanswered queries for this process. However, there are several shreds of evidence that suggested, eggplant domesticated from *S. insanum* through multiple and independent domestication process naturally spread in tropical Asia from the Philippines to Madagascar [24] in several centers of domestication [25].

Interestingly, the evidential proof of cultivation of eggplant found both India and China equally. Together with archaeological evidence about eggplant showed there was the utilization of eggplants started earlier in India than China, with a subsequent additional and independent center of domestication in the Philippines [25]. During the 8^th^ century, the eggplant was distributed eastward to Japan and westward via silk route into western Asia, Europe and Africa. It was introduced in the Americas in the 14^th^ century by the Europeans [26].

Likewise, the New World has emerged as the center of primary diversification “hotspot” for the genus ‘*Solanum*’ because of higher species diversity. Still, some authors recognized Australia as a secondary center of diversity [27]. Contrary to expectation, Echeverria-Londoño et al. [28] reported the rate of diversification is faster in the case of the Old-World clade of spiny solanums, despite its lower numbers of species found as compared to the New World clades. Based on the dated phylogeny, Särkinen et al. [29] reported explosive diversification in the Old-World regions, specifically in Australia. They also hypothesized, there was a long-distance dispersal event approx. ca. 6 Mya, followed by there was a rapid expansion of new niches gradually opened up by the spread of dry forest habitat types. In light of these findings, prospects and analysis of the relationships of Australian spiny solanums will undoubtedly help to explain its patterns of diversification and expansion in the region. Several abiotic stresses feature by Australian solanums definitely will have a great interest to eggplant breeders in the coming future.

## 3. Breeding for Eggplant

The conventional breeding approaches to improve crop plants are the introduction, mass selection, pure-line selection, pedigree selection, single seed descent, bulk method, and backcross method. According to the situation and objectives, combinations of approaches have been found a valuable strategy. However, a lot of efforts are made in the direction of breeding for earliness, decreased fruit bitterness, and reduced prickliness. Tremendous work has been done in the few decades by plant breeders on the adaptation of eggplant and its yield improvement under the greenhouse conditions. Loss due to insect pests and diseases demands eggplant breeding for biotic stress resistance without compromising for yield [30,31]. Similarly, several quality factors are also considered, namely fruit shape, fruit color, plant prickliness, fruit palatability, and glossiness [32].

### Wide Hybridization, Embryo Rescue

**Figure 1:**
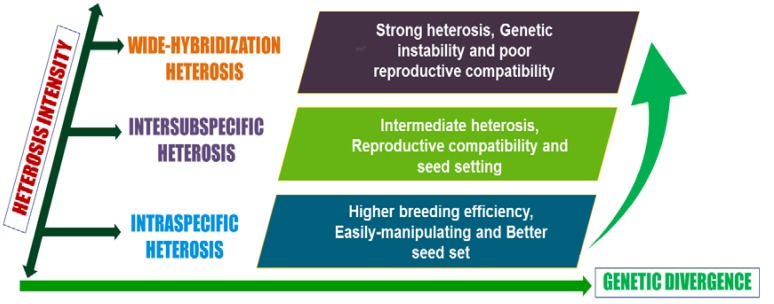
Classification and Features of heterosis exploitation.

Wild relatives of eggplant come up with the excellent potential for their use in breeding programs. Instead of tremendous diversity and beneficial alleles for biotic and abiotic stress present in wild relatives, there has been little progress made on in its overall improvement [33]. One of the significant obstacles to the use of alleles of wild species into cultivated eggplant the is lack of a genome sequence database [34]. However, the recently mapping population developed from several crosses between eggplant and its wild relatives [35]. Species come under secondary or tertiary gene pool can be used for crossing with cultivated ones by the use of embryo rescue with varying rate of success [36]. It has been noticed that the degree of cross-compatibility is variable among the cultivated eggplant and wild relatives. It was also determined that the introgression of genes or segments of chromosomes from the wild species to cultivated species might be more comfortable in some cases. For improvement of eggplant, identification of best potential wild species for distance crosses depends upon extensive morphological phenotyping of the parents, F_1_s and their advancing progenies (Figure 1) (Table 1) [35].

## 4. Heterosis Prediction

To evaluate a large number of lines, breeders have to make hundreds of test crosses and estimate F_1_ to identify best hybrids in respect of yield and other quality traits. Handling several crosses simultaneously is not an easy task. Because of that, it is crucial to identify the superior crosses with high heterotic potential. There are several ways for the prediction of heterosis, viz., *per se* performance of parental lines, combining ability, mitochondrial complementation and genetic diversity, multivariate analysis of morphological traits, coefficient of parentage and isozyme and molecular marker-basedanalysis [38]. Inadditiontotheseapproaches, inrecentstudies, geneexpressionisusedtopredictheterosis. Generally, heterosis prediction is estimated with *per se* performance, analysis of genetic diversity, combining ability analysis of parental lines. There are several reports in different crops found contrasting conclusions about the effectiveness of *per se* performance for the prediction of heterosis. Contrarily selection of better parents which is based on *per se* performance found to be very useful in case of *Triticale* for several traits except for grain yield [39]. However, in tropical maize under extreme moisture stress condition, the performance of hybrid progenies can be predicted to some extent based on *per se* performance of their inbred parents [40].

Interestingly, there is absolutely no association located amongst *per se* functionality of parental lines and heterosis in F_1_ hybrids in maize [41] and sugarcane [42]. For that reason, the prediction of heterosis only based on the *per se* performance of parents is not an essential indicator of heterosis. There is a necessary and robust correlation located amongst genetic distance and heterosis in rice [43], maize [44], wheat [45], sunflower [46] and rapeseed [47]. Moreover, the omics-based approaches have excellent potential for the prediction of heterosis. Zenke-Philippi et al. [48] reported mRNA transcription profiles are a terrific selection to DNA markers for the prediction of hybrid performance. Even so, added investigation obtaining more massive data sets is essential to investigate the feasibility of selection prediction models.

The first results of the prediction of hybrids by using mRNA transcriptomics were determined by Frisch et al. [49] and with the help of regression-based methods by Fu et al. [50].General combining ability (GCA) or testcross performance-based hybrid prediction is a particular case of hybrid prediction and is regularly applied in eggplant breeding and hybrids development [51,52,53]. In this context, sometimes, the metabolites are also used as predictors [53]. Vacher et al. [54] found that additive effects in combination with intricate patterns can explain most or all the heterosis seen in typical F_1_ hybrids. Although, heterosis is a genome-wide phenomenon covering the network of genes and their proteins leading to depictions in the form of the phenotype by changes and modifications in the plant metabolism. Still, there is a vast scope in advances in the expression-based prediction of heterosis, which provides new avenues for the same.

## 5. Genetic and Molecular Basis of Heterosis

According to the dominance hypothesis, the independent set of deleterious alleles accumulates over time and illustrates their expression in the homozygous recessive condition during the inbreeding process [55,56]. The dominant alleles coming from one parent complements its counterpart, minor allele from the second parent, ultimately gives better phenotype. However, according to the over-dominance hypothesis, allelic interactions that stimulate heterozygous loci expression in hybrids [8]. Intra-allelic interaction having a significant role in over-dominance where the presence of multiple alleles gives excellent performance than homozygosity for either of alleles. If over-dominance is a major cause of heterosis, breeding methods that maximize heterozygosity will result in the best performance. Whereas on the other hand, if dominance or epistasis is the primary cause of natural or breeding populations—as well as individuals behaving similarly to hybrids—by fixing up for favorable alleles.

Hallauer et al. [57] have addressed this issue from the early to mid-1900s by analysis of variance components. Moreover, Moll et al. [58] observed that estimates of variance could be influenced by linkage. Specifically, in a condition, when negative and positive alleles were linked in the repulsion phase. None of these two hypotheses describes the effect of interactions between non-allelic loci. Epistasis is the inter-allelic interaction between two or more genes. According to Fiévet et al. [59], epistasis can mimic over-dominance. Due to complexity, the role of epistasis with heterosis was not that fully understood. Afterward, the complex nature of the biological process and its networks which signify polygenic traits become understandable [6]. The role of epistasis concerning heterosis remains challenging to understand.

In early studies because of limited experimental size and computational capacity, estimates of the epistatic variance of heterosis were minimal. Afterwards, generation means analysis provided some of the first compelling evidence of the role of epistasis in hybrid performance. Still, the role of epistasis concerning heterotic as well as non-heterotic trait performance remains intriguing and perplexing. Several diverse, complex pathways are interacting themselves to produce phenotypes in individuals.

Genetic epistasis not only has interactions between several molecular pathways but also has allelic variations within specific interacting pathways, which result in significant statistical interaction. However, QTL mapping studies show interest in interaction effects for specific developmental, architectural and biochemical traits. Although heterosis is more significant for trait-like yield, which highly complex [60,61,62]. All of these mechanisms could, and probably do, lead to heterosis. However, the debate continues over, which is more important because genetic effects are difficult to access. From the point of advance molecular genetics, there are several basic questions which remained unanswered for heterosis in plants. Although over time, the loci governing heterosis are becoming more evident. However, it is seen that a few genes show over-dominance effects at the heterozygous state. Huang et al. [63] observed ‘*IPA1*’, the gene showing over-dominance effects controlling several yield components. However, the genes which show over dominance effect for a specific component, the heterotic effects might be due to an optimal level of gene expression. Although its mechanism is still unclear at gene regulation level. Further study needs to dissect the heterosis phenomenon, which relies on a molecular mechanism based on physiological and developmental biology approaches. A recent review by Liu et al. [64] provide insights to recent advances on genetic and molecular components of heterosis in plants.

## 6. Heterosis in Eggplant

Heterosis is a phenomenon that appears in the F_1_ generation, depicts itself by rapid growth and development, higher productivity, greater vitality, resistance, and uniformity. In fact, in the case of eggplant, exploitation of heterosis or hybrid vigor has become an important tool for overall improvement in eggplant reported from the very beginning [65,66]. There is an immediate increase in size and weight of eggplant due to increment in embryo size reported by Kakizaki [9]. Considerable hybrid vigor was observed as early as in 1892 by Munson [67] in the USA. As well as in Japan by Nagai and Kida [68]. Despite economic as well as nutritional importance, breeding efforts in eggplant are still limited, because of that, its production is lower in comparison with other solanaceous crops [69].

First and foremost, the objective of eggplant is to develop high-yielding varieties, mostly F_1_ hybrids, having a high degree of stress tolerance level [70]. Although, it is not possible to breed a single variety, having better adaptability for multiple environments as well as to meet consumer preferences. Therefore, breeding of suitable locally adapted hybrids with preferred fruit characters having high yield and adaptation is mainly achieved through heterosis breeding (Table 2) [71,72]. A few decades back, the concept of heterosis was based on the biochemical and physiological parameters [73]. But the recent findings in molecular genetics have confirmed that the actual cause and effect of heterosis is purely genetical [74]. Generally, evaluation of elite-breeding lines as parents and its first filial generations (F_1_s) to detect heterotic potential becomes a routine practice in heterosis breeding [75]. In eggplant, Mistry et al. [69] found significantly positive heterobeltiosis for fruit volume, fruit length, and fruit yield per plant, which reflect the hybrid vigor can be used on a commercial scale for these traits. The selection of parents is a very critical step that reflects the performance of hybrids for hybrid breeding programs [76]. The parents must have good general combining ability, as well as the specific combining ability [77]. Furthermore, till now there is no finding in which diallel analyses coupled with genotyping by molecular markers to examine the reliability and feasibility of molecular markers for the selection of better parents which reflect into good hybrids in eggplant [4].

Although Rodríguez-Burruezo et al. [78] reported, there is a positive correlation between genetic distances based on AFLP molecular markers with the heterosis of hybrids as well as the yield of hybrids. Based on their conclusions, these authors used only ten hybrid combinations based on local Spanish varieties. Although the results can be contrasting based on the founding parents of the hybrids and molecular markers employed [79,80,81]. For fruit yield, Singh and Kumar [82] reported the highest heterosis (162.5%) over the better parent. Likewise, Saha et al. [83] found maximum heterobeltiosis for several branches per plant and plant height was 48.45 and 26.4 percent, respectively. Das and Barua [84] determined the majority of the crosses of eggplant demonstrated a highly significant level of heterosis for yield and contributing traits, similar findings were reported by Kaur et al. [85]. Similarly, Patil et al. [86] found heterotic effects due to fruit weight (150.27 g), seed percent (9.57%), length of fruit (13.22 cm) and yield of fruits (3.19 kg/plant), gives a clear indication for the exploitation of heterosis at commercial level.

## 7. Prospects of Male Sterility in Eggplant Heterosis

In most cases, the goal of the breeder is to develop improved high yielding cultivars. However, it has seen that from the last few decades, the popularity of hybrid cultivars has been increased dramatically. Cytoplasmic male sterility (CMS) is a helpful phenomenon for hybrid seed production in a large variety of crop species. CMS in plants is a sort of sterility caused by specific nuclear and mitochondrial interactions. It is a maternally inherited trait that enables breeders to exploit the hybrid vigor [92]. Nagai and Kida [68] first reported quantitative traits in eggplant hybrids and observed that heterosis was expressed in total yield, plant height, number of branches, early maturity, number of fruits per plant, number of spines on the pedicel, and fruit weight. With the increasing economic importance of eggplant, the use of male-sterile lines in eggplant breeding is increased to produce hybrid seeds. New evidence from recent studies on male sterility’s molecular mechanism provides a valuable roadmap for heterosis breeding programs. Although, male sterility molecular mechanism has been studied in several crops but is still poorly understood in the eggplant. Several genic [93], cytoplasmic [94,95] and genetically engineered [96,97] male sterility system have been developed in eggplant. Genic male sterility is of minimal practical application due to its mode of utilization and maintenance [98]. Besides, there is some evidence that reports genic male sterility expression in eggplant is influenced by abiotic factors [99].

However, in many countries, the cultivation of genetically engineered crops is not approved [100]. Alternatively, the maintenance of CMS and its use in hybrid seed production is essential due to the maternal inheritance of the male character and its mode of fertility restoration [98]. Bentolila et al. [101] reported, in many instances, that male sterility can be restored by nuclear-encoded fertility restorer (Rf) gene. The key role in the nuclear genetic regulation of CMS is controlled by Rf genes, which are important for the restoration of male fertility after interaction with the CMS-inducing cytoplasm. Therefore, the development of a robust CMS system with appropriate Rf-genes is an effective utilization in the hybridization system. Yoshimi et al. [102] reported the variation in the flanking DNA sequences of five mitochondrial ATP and COX genes as the causal genes for each CMS type variation in the eggplant. Recent findings suggested that eggplant parent genotype can influence both the CMS expression and fertility restoration [10]. With the advancement in genomics and transcriptomics studies, the effort has been made ease in identifying molecular marker linked to the Rf-genes which promote the integration of Rf-genes in various eggplant genetic backgrounds. Recently, comparative transcriptome analysis has been performed to identify the MS-related genes and pathway in the eggplant of the male sterile line and its maintainer line [103]. The finding of this study provided insight into key genes and pathways associated with eggplant male sterility, which provided a primary basis for further research on fertility and anther development.

## 8. Epigenetic Regulation of Heterosis in Eggplant

Recently, enormous progress has been made in terms of the role of epigenetic regulation in crops as a new, timely crop breeding tools. The mixture of divergent maternal and paternal genomes inside the very same nucleus may lead to genomic instability, epigenetic alterations and altered gene expression, which can eventually trigger phenotype alterations inside the hybrid. Changes in the gene expression behavior fall into the epigenetic changes independent of changes within the DNA sequence [104,105].Growing evidence suggests that epigenetic variables play a vital part in heterosis [106,107]. Heterosis has been associated with many interactive attributes, including alterations in gene expression, metabolic activity and epigenetic regulation [108,109,110,111]. In plants, biogenesis of an important group of 24-nucleotide siRNAs relies on RNA-dependent RNA polymerase two (RDR2), RNA polymerase IV (Pol IV) [112,113] and endonuclease DICER LIKE 3 (DCL3) [114,115]. Such siRNAs interact with ARGONAUTE4 (AGO4) and resulting in gene silencing and/ or RNA-directed DNA methylation (RdDM) at target sites [114,115,116]. A lot of siRNAs originate from transposable components and repeats which have already been located diverged amongst species.

**Figure 2:**
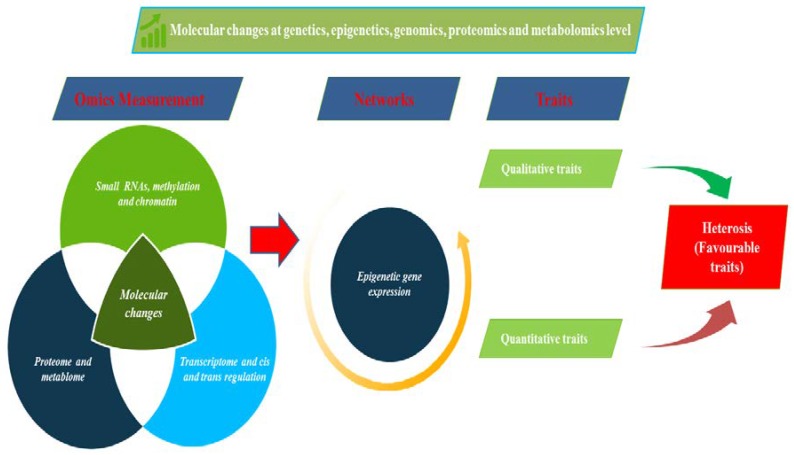
Molecular changes at genetics, epigenetic, genomic, proteomic, and metabolic levels leading to favorable heterosis traits.

Apart from, siRNAs showed expression alterations in F_1_ hybrids of rice [117] and maize [118] compared with their parental strains. miRNA-encoding loci are transcribed by RNA polymerase II [119] and precursor transcripts are processed in plants [120] by DICER LIKE 1 (DCL1). In the case of RILs (cultivated tomato and its wild relative), some siRNAs and miRNAs are associated with transgressive RILs phenotypes but are absent from parents [121]. These studies showed a part in vigor hybrid phenotypes for miRNAs and siRNAs. In each of the hybrids, genome-wide methylation, expression of sRNAs and gene were studied; its parents showed variation among them. DNA methylation happens at CG, CHG and CHH sites on cytosines in plants (exactly where H = A, T or) [114]. The amount of DNA methylation located the adjust in intraspecific *A. thaliana* hybrids [122,123] and rice [124] are related to parental plants. In reciprocal *A. thaliana* F_1_ hybrids [122] enhance in methylation at CG sites, from 18%–26% to 36%–37% and inside the amount of the F_1_ hybrid of CHG and CHH is somewhat larger than in the parents. The extent of alterations in methylation in hybrids depends on parental differences; far more changes are observed in DNA methylation with greater heterogeneity. In hybrids and polyploids the epigenetic and epigenomic variations are related to parent-of-origin or imprinting effects [125]. These effects are connected to a group of Pol IV dependent siRNAs (p4 siRNAs), depending on maternally transmitted of these siRNAs [126]. Maternal expression of siRNAs is negatively associated with the AGAMOUS, LIKE (AGL) encoded genes Type I MADS-box transcription factors expression, in the endosperm, which is involved in seed size regulation [127]. Variations in siRNA and patterns of methylation observed between parents [122,127,128] are expected to form allelic methylation in hybrids through RdDM and/ or expression of allelic patterns. Epigenetic modification like RdDM and its histone marks on parental alleles may possibly give ‘memory’ resulted in parental origin effects on gene expression [129]. Epigenetic variants of interest may perhaps even currently exist, but not yet remain described. Comparative transcriptome studies among hybrids and their parents showed a wide array of genes had altered expression levels when compared with the expression of their parents [107,129]. Various studies of transcriptomics, proteomics, and metabolomics in this direction provided insights into regulatory components of hybrid vigor phenotypes (Figure 2).

To higher recognize the genetic basis of wild associated adaptive phenotypes becomes a prominent purpose in eggplant breeding. Inside the era of high-throughput next-generation sequencing (NGS), it becomes simple to develop molecular markers for diversity evaluation, genetic mapping and candidate gene discovery. Molecular markers generated from higher throughput sequencing (especially employing RADseq and genotyping-by-sequencing methods) give the way of building of gene mapping and to study genetic diversity occurs inside accessions of eggplant and also between eggplant and its wild relatives.

## 9. Application of Transgenic Approaches and Genome Editing

To feed the growing population under the situation of global climate change, the transgenics with biotic or abiotic stress resistance genes taken from other organisms is a promising tool [130], although the perception of people is mixed, and even in some cases strongly negative. Some part of negativity is coming because of the lack of published work regarding the consequences to other related or unrelated organisms of growing a genetically modified (GM) crop. These non-target effects, like the effects of GM crop on soil microbiome [131] and different food chains [132], remain understudied. Likewise, in present scenarios, the breeding paradigm is shifting from conventional to introgression breeding approaches by doing wide hybridization, embryo rescue, and genetic modification as well by using different genetic engineering tools. The purpose behind this is to introgression of abiotic and biotic stress resistance genes by making distance crosses and by using genetic engineering tools. GM eggplants with the *Cry1Ac* toxin derived from *Bacillus thuringiensis* popularly known as *Bt* Brinjal. In future, locus-specificity epigenome editing methods such as *CRISPR-Cas9* [133] might be used for the identification and development of epigenetic variants. Inevitably, managed manipulation of gene expression from ‘heterotic’ pathways and genes that led to the variations into the magnitude of heterosis, may perhaps be of importance in crop production systems.

## 10. Conclusions and Future Perspective

Heterosis can now be regarded as a result of genome interactions, leading in complex modifications at genetic, epigenetic, biochemical, and regulatory network levels. In recent decades, several efforts have been performed in heterosis related research. In the field of heterosis research, new technological advancements have facilitated a better understanding of the heterosis phenomena. Therefore, most of the studies involving crossing in eggplant end up in the estimation of the extent of heterosis. Although eggplant has undergone an enormous selection pressure for the trait especially higher yield. Moreover, even when developing hybrids for disease and insect pest resistance yield is generally not compromised.

**Figure 3:**
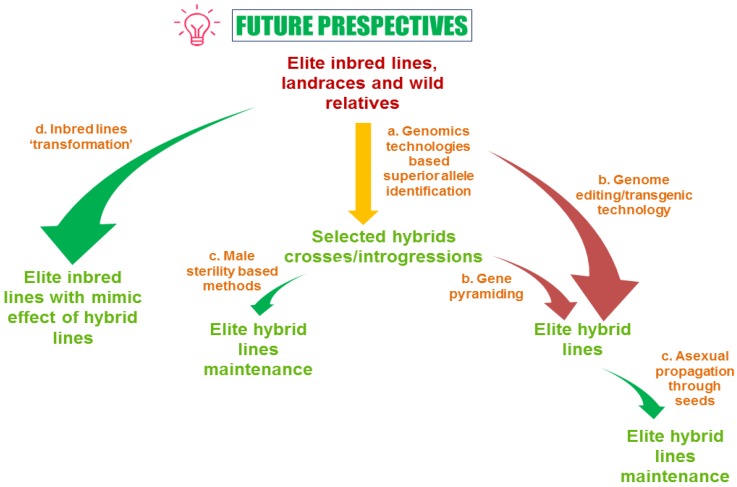
Future perspectives on heterosis breeding.

Furthermore, to keep with growing market demand of eggplants, the hybrids are desired by the farmers because of their higher yield potential. But the hybrid performance depends on the parents (inbreds) used in the hybridization program. Generally, distinct inbreds lead to more heterotic hybrids. Whereas in eggplant, this may vary as in a recent study, it was pointed out that SNPs based genetic distance determined for the morphological and the biochemical traits it does not significantly affect the heterosis in eggplant. Heterosis exploitation is significant in eggplant to obtain traits like higher vitality, better growth and development, insect and pest resistance and uniformity. The first report of heterosis in eggplant was presented in the early 19^th^ century. With a relatively less cost of hybrid seed production in eggplant and the availability of male-sterility mechanism, the development of new hybrids is straight forward. The combination of data from various omics approaches like; transcriptomics, epigenomics, proteomics and metabolomics can be used in the future for mapping and cloning of complex heterosis related genomic regions through map-based cloning may allow identifying multiple key-related genes of heterosis (Figure 3). In this context, a significant challenge is to accurately track and quantification of the diverse heterotic phenotypes that contribute to nearly all heterotic traits. We believe that understanding the connections between different studies over the coming years, will clear the association between the genetic hypotheses and molecular actions leading to heterosis. Thus, recent advances in new genetic and genomic tools will drive forward the understanding of complex interaction between genome structural organization and expression of genes.

## Figures and Tables

**Table 1:** Heterosis in the eggplant using wild relatives as the male parents taken from Kaushik et al. [35,37].

Descriptors	*S. insanum*	*S. anguivi*	*S. incanum*	*S. lichtensteinii*	*S. linnaeanum*	*S. tomentosum*
Plant height (cm)	16.7 ± 4.6	34.4 ± 7.1	36.8 ± 11.3	38.1 ± 4.4	2.3	23.3 ± 4.2
Stem diameter (mm)	10.5 ± 4.3	10.4 ± 3.8	29.1 ± 11.0	39.8 ± 10.3	−18.7	23.8 ± 3.8
Leaf prickles (upper surface)	155.1 ± 34.5	260.0 ±173.9	733.3 ± 100.0	144.4 ± 92.9	100	800.0 ± 800.0
Leaf pedicel length (cm)	39.7 ± 6.5	22.5 ± 7.8	19.5 ± 2.7	24.9 ± 9.2	−13.3	56.3 ± 23.9
Leaf blade length (cm)	24.9 ± 4.1	22.2 ± 5.5	47.6 ± 6.6	30.6 ± 6.3	3.9	22.8 ± 1.6
Leaf blade width (cm)	27.7 ± 4.5	38.2 ± 9.5	67.7 ± 9.6	41.7 ± 8.5	7.1	22.4 ± 14.0
Number of flowers per inflorescence	70.1 ± 16.0	75.9 ± 16.3	21.0 ± 9.4	42.7 ± 15.7	−1.8	87.7 ± 35.5
Corolla color	15.9 ± 4.3	−2.5 ± 4.6	19.2 ± 3.0	16.2 ± 4.8	7.5	−0.1 ± 8.6
Number of petals	1.3 ± 2.1	−4.8 ± 1.6	−4.4 ± 2.4	−2.2 ± 3.4	−3.2	−1.0 ± 1.0
Fruit weight (g)	−5.5 ± 6.9	−98.2 ± 0.3	−86.6 ± 2.8	−89.4 ± 1.5	−89.9	−98.6 ± 0.3
Fruit calyx prickles	32.9 ± 25.2	−100.0 ± 0.0	27.1 ± 42.4	56.9 ± 27.6	80	29.1 ± 104.1
Dry matter (%)	−2.9 ± 3.6	−35.8 ± 6.6	12.4 ± 12.8	−16.6 ± 7.4	41.6	−31.6 ± 14.1
Total phenolics (g/kgDW)	−23.3 ± 5.2	16.9 ± 10.2	52.5 ± 17.1	19.9 ± 9.3	21.8	73.9 ± 25.1
Chlorogenic acid(mg/g)	−21.8 ± 5.7	76.5 ± 17.8	1.5 ± 6.9	11.5 ± 3.5	4.9	38.5 ± 8.2
Polyphenol oxidase activity	16.5 ± 17.8	65.6 ± 27.9	50.3 ± 81.1	23.8 ± 32.5	−13.7	187.3 ± 42.1
Degree of browning	−16.8 ± 12.8	85.9 ± 20.2	224.6 ± 48.1	138.6 ± 26.6	143.9	−9.1 ± 8.5

**Table 2:** Heterosis in cultivated eggplant for important morphological and fruit biochemical traits.

Trait	Types of Crosses	Range	References
Fruit Yield (q ^ha-1^)	di allel,	50.48–62.20	[87,88]
Plant Height (cm)	di allel, Line × Tester, F_1_ Crosses	6.09–57.77	[69,87,88,89]
Fruit Yield/ Plant(kg)	half diallel, Line × Tester, F_1_ Crosses	28.95–63.54	[69,88,90,91]
Number of Fruits/Plant	half diallel, Line × Tester, F_1_ Crosses	14.56–158.90	[69,88,90]
Fruit Weight (g)	F_1_ Crosses	19.8	[69]
Fruit Length (cm)	Line × Tester, F_1_ Crosses	21.81–47.08	[69,90]
Fruit Girth (cm)	Line × Tester, F_1_ Crosses	19.15–29.05	[69,90]
Ascorbic acid content (mg/100)	half diallel	22.39	[91]
Total Phenolic Content (mg/100)	half diallel	7.97	[91]
